# Molecular characterisation of fowl adenovirus type 7 isolated from poultry associated with inclusion body hepatitis in Poland

**DOI:** 10.1007/s00705-017-3240-5

**Published:** 2017-02-03

**Authors:** Jowita Samanta Niczyporuk

**Affiliations:** Department of Poultry Viral Disease, National Veterinary Research Institute, Partyzantów 57 Avenue, 24-100 Pulawy, Poland

## Abstract

The fowl adenovirus field strain FAdV-JSN-5/10j (GenBank accession number KP879219) was isolated from the intestine of a 7-week-old chicken diagnosed with inclusion body hepatitis and simultaneously with Marek’s disease, and for that reason, it was chosen for molecular study. It was identified as fowl adenovirus genotype 7 (species *Fowl aviadenovirus E*) based on nucleotide sequence analysis of the loop L1 region of the hexon gene. Nucleotide sequence alignment of this strain, FAdV-7 reference strains B-3A ATCC VR-832 (AF339922) and YR36 (AF508955), and eight additional FAdV-7 field strains confirmed its classification as FAdV-JS-5/10j and showed that these viruses are very similar to each other. Additionally, we described mutations and their influence on the amino acid sequence, nucleotide composition, and relative synonymous codon usage. Immunofluorescence of cell cultures infected with 10^4.5^ TCID _50_ per 0.1-ml dose of the FAdV-JSN-5/10j strain demonstrated the presence of a cytopathic effect. Infection of fowl with adenoviruses raises concerns for poultry production, and thus, the efficient detection of adenovirus infection is crucial. This is the first attempt to describe the molecular characteristics of FadV-7 strains isolated in Poland.

## Introduction

Viruses belonging to the family *Adenoviridae* are divided into five genera. Fowl adenoviruses (FAdVs) belong to the genus *Aviadenovirus*, which is further divided into 12 species: *Fowl aviadenovirus A-E*, *Duck aviadenovirus B*, *Falcon aviadenovirus A*, *Goose aviadenovirus A*, *Pigeon aviadenovirus A*, and *Turkey aviadenovirus B-D*. FAdVs are divided into twelve serotypes (1-8a-8b-11). Adenoviruses are large, non-enveloped viruses that contain a dsDNA genome [17, 28]. A significant percentage of the different FAdV serotypes cause disease in poultry [1, 8]. Avian adenovirus serotypes 6, 7 and 8 have been reported to cause inclusion body hepatitis (IBH) in Australia, New Zealand, and other parts of the world [20]. Various researchers from India have reported the presence of FAdV serotypes 2, 5, 6, 7 and 12 in addition to serotypes 4 and 8 [22]. FAdVs were detected in liver samples obtained from broiler chicken flocks with IBH or hydro-pericardium syndrome (HPS) by PCR using the hexon gene [22]. In Pakistan, many FAdV strains of serotype FAdV-4 have been isolated from broiler flocks with HPS [27].

Thirteen avian adenovirus genome sequences are mentioned in the ninth report of the ICTV. Much is known about the genome organization of adenoviruses, but not all of the genes have known functions [11, 19].

The complete DNA sequence and genomic organization of the FAdV-1serotype strain CELO has been described by Chiocca et al. [3], Washietl and Eisenhaber [29] and, Xu et al., [30]. The strain became a reference strain for the genus *Aviadenovirus*. However, there are noteworthy differences between the genome sequence of the CELO strain and sequences of adenovirus strains representing other serotypes [4, 12, 13, 15, 18]. The structure of the adenovirus genome and the location of the hexon gene and HVR1-4 (hypervariable regions) have been described [21, 29, 30].

Domańska-Blicharz et al. [6] described the molecular characteristics of an FAdV-A CELO isolate found in Poland, but there has been no report of the presence of the FAdV-7 serotype in Poland. The aim of this study was to determine the nucleotide sequence of a portion of the genome of a field isolate from a sick chicken and to determine the relative synonymous codon usage (RSCU) in the loop L1 region of the hexon gene, as well as to compare it with two sequences of reference strains and field strains obtained from the GenBank database.

## Materials and methods

### Chicken embryo fibroblast (CEF) cultures

CEF cultures were prepared from 9- to 11-day-old SPF chicken embryos (Lohman, Germany) according to the standard procedure. Eagle’s growth medium (MEM) was used with addition of 10% foetal bovine serum and 1% antibiotic mixture (antibiotic-antimycotic, Gibco, U.K.). The maintenance medium consisted of MEM with 1% antibiotic-antimycotic mixture. A monolayer of CEF culture was obtained after 24 h incubation at 37.5°C.

### Adenovirus field strain

The JSN-5/10j strain (accession number KP879219) was isolated from 7-week-old chickens infected with Marek’s disease virus (MDV) and was associated with a clinical field case of inclusion body hepatitis (IBH). Clinical signs characteristic of IBH and gross lesions in the liver and kidneys of dead chickens were observed in the examined flock. The liver was swollen and friable with multifocal areas of necrosis and petechial haemorrhages. The mortality rate in that flock was approximately 10%. The isolated strain was specifically linked to the disease outbreak. The third passage of the strain was used for the infection of CEFs.

### Virus reference strains

The reference strain, belonging to the serotype ATCC FAdV-7, was obtained from a commercial company (Charles River, USA) and was used as a positive control in cytopathic effect (CPE) assays, immunofluorescence (IF), and real-time PCR. Two sequences of reference strains, FAdV-7: B-3A ATCC VR-832 (AF339922) and YR36 (AF508955), and eight field sequences derived from the GenBank database (NCBI) were used for nucleotide and amino acid sequence comparisons.

### Virus replication

Homogenates from internal organs of sick chickens were prepared as a 1:1 dilution in Eagle’s medium containing a 1% antibiotic mixture (antibiotic-antimycotic, Gibco, UK), and then filtered through a 0.45-µm Millipore filter (Minisart, Sartorius, Germany). Filtered homogenates and lyophilisates were used for infection of CEFs. CEF cultures were incubated at 37°C for five days in the presence of 5% CO_2_. The appearance of CPE characteristic of FAdV infection was monitored daily using a microscope. The third passage of each strain was kept at -20°C for the next step of the study.

### DNA extraction

Total DNA of reference strain ATCC FAdV-7 and the field isolate FAdV-JSN-5/10j was extracted using a DNA Mini Kit (QIAGEN, Germany) according to manufacturer’s procedure. DNA was isolated directly from CEF cultures infected with field and reference FAdV-7 strains as a positive control. DNA was also extracted from uninfected CEF cultures as a negative control. DNA samples were then stored at -20°C for the next step of the study.

### Determination of tissue culture infection doses (TCID_50_)

The TCID_50_ values of field and reference strains were determined using 24-well plates (Thermo Scientific, USA) coated with CEF cultures (18-24 h). CEFs were infected with tenfold dilutions of virus stocks from 10^−1.0^ to 10^−7.0^ in triplicate for each dilution and three wells for the negative control. The plates were incubated at 37.5°C with 85% humidity in an atmosphere of 5% CO_2_. CPE was observed using a microscope (Zeiss HXP 120, Germany) on a daily basis. After 6 to 7 days of incubation, the results were read according to the Reed and Muench model, and the TCID50 value was determined.

### Immunofluorescence assay (IFA)

CEF cultures were infected with the third passage of the JSN-5/10j and ATCC FAdV-7 strains. When CPE was observed after 5-6 d.p.i., CEFs were covered with 90% acetone (POCH, Poland) cooled to -20°C. After 30 min, the acetone was removed, and the plates were allowed to dry for the next 24 h. The CEFs were washed three times with PBS buffer (Biolab, Poland), followed by the addition of 500 µL of blocking mix: 1x PBS, 5% bovine serum, and 0.3% Triton X-100. The plates were incubated for 1 h at 18-24°C, the blocking mix was removed, and 500 µL of mouse primary FAdV antibody (Charles River, USA), diluted 1:100 in PBS, was added. After incubation at 37°C for 18 h, the plates were washed three times with PBS (Biolab, Poland), and a 1:200 dilution of a secondary rabbit antibody against mouse IgG_1_ conjugated with fluorescein isothiocyanate (FITC) (Serotec, Germany) and incubated at 18-24°C for 2 h in the dark. The fluid was removed and the plates were washed three times with PBS buffer. The cells were viewed using a fluorescence microscope (Zeiss, Axio Observer D1, Germany). The presence of fluorescent cells of different sizes indicated a positive result in the IFA. CPE was recorded using a camera (Axiocam MRm, Germany).

### Real-time PCR for the identification of the adenovirus hexon gene

The sequences of nucleotide primers specific for adenoviruses were as follows: FAdV JSN (sense primer), 5’ AATGTCACNACCGARAAGGC 3’; FAdV JSN (antisense primer), 5’ CBGCBTRCATGTACTGGTA 3’; TaqMan probe JSN RT, 5’ AATCCCTACTCGAACACCCC 3’. The predicted size of the product was 93 bp, as reported previously by Niczyporuk [23].

### Sequencing and molecular analysis

The amplification product from the JSN-5/10j loop L1 region of the hexon gene was purified using NucleoSpin Extract II (Marcherey-Nagel, France) and then sequenced by GENOMED (Poland) using a GS FLX/Titanium sequencer (Roche, Switzerland). Sequence comparisons were performed by alignment of the nucleotide sequences of the amplified fragments originating from the hexon gene with fowl adenovirus serotype 7 reference sequences B-3A ATCC VR-832 (AF339922), and YR36 (AF508955) obtained from the GenBank database (NCBI). A phylogenetic tree was generated by the neighbor-joining method using the p-distance method (on 1000 bootstrapped datasets). Sequence analysis was performed using the software MEGA6, Geneious7, and BLAST.

### Analysis of nucleotide and amino acid sequences

The sequences of strain JSN-5/10j and FAdV-7 reference and field strains were assembled using the MEGA6 program. To confirm the correctness of the assembled sequences, they were compared to reference, field and JSN-5/10j strains. The predicted amino acid sequence of FAdV genome fragment containing loop L1 of the hexon gene was determined using Geneious7.

## Results

### Infection of CEF Cells

**Fig. 1:**
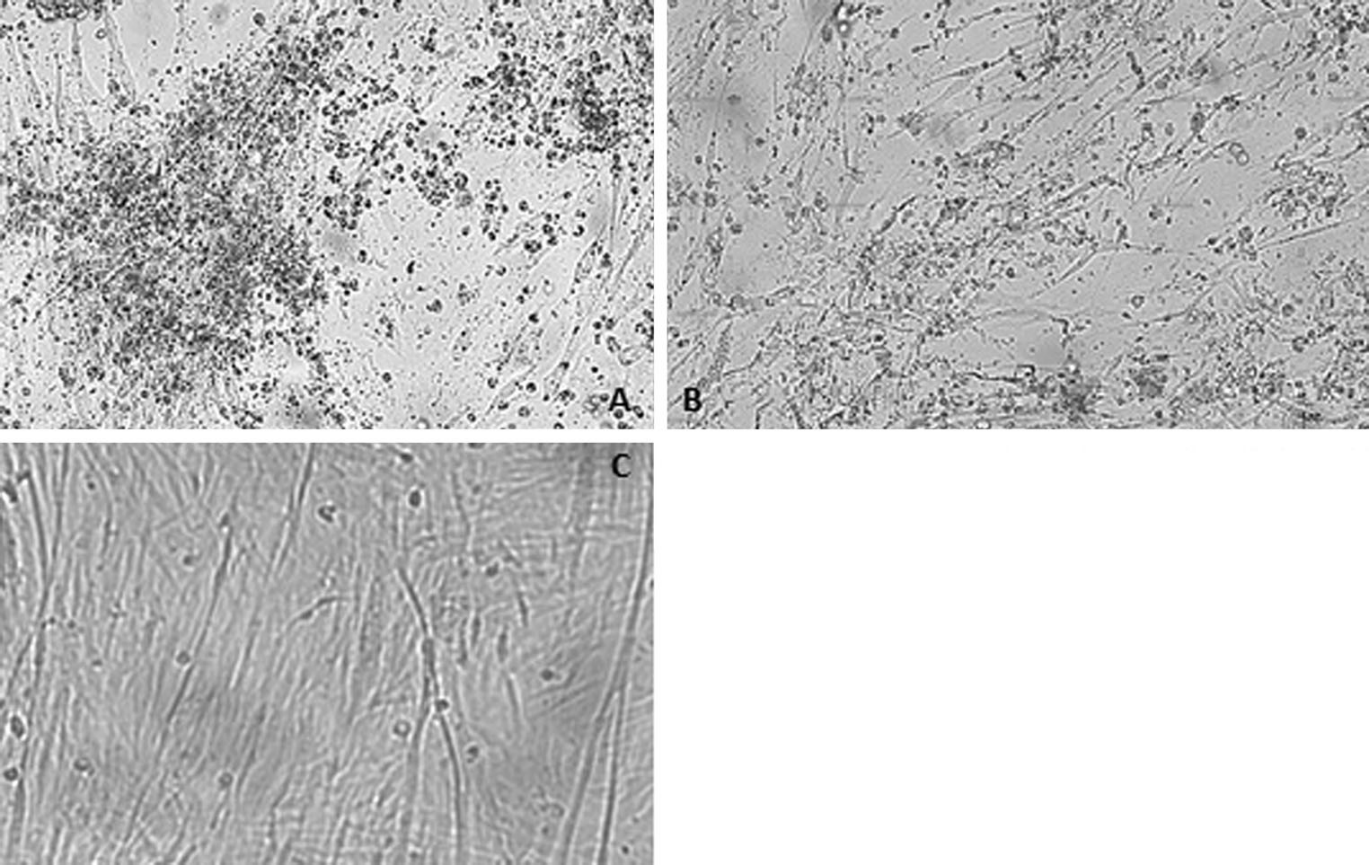
**A**. Cytopathic effect of reference strain FAdV-7 Charles River, US. **B**. Cytopathic effect of -JSN-5/10j strain IIIp, 96 h of incubation. **C**. Uninfected CEFs, K- negative control

Monolayers of CEF cultures were infected with JSN-5/10j and reference strains. Three passages (96 h for each of them) were conducted. During the third passage, the first CPE was recognized at about 18-24 h postinfection. Infected cells were bigger and rounder than uninfected cells, and they were filled with granules. In the subsequent days, the number of altered cells increased, and the cells covered the surface of the bottles. Changes in the pH of the medium were observed, and this was also a factor contributing to damage of the cells. The observed CPE differed in intensity. Strain JSN-5/10j caused CPE, which was compared to the CPE obtained with ATCC FAdV-7, which was used as a control. The cytopathic effects induced by the ATCC FAdV-7 and JSN-5/10j strains are shown in Fig. 1A-C.

### TCID_50_ determination

TCID_50_ of the examined strains in CEF cultures was determined from the third passage. In the case of the ATCC FAdV-7 strain, it was 10^5.0^ TCID_50_/0.1 ml, and for the JSN-5/10j strain, it was 10^4.5^ TCID_50_/0.1 ml.

### IFA determination

**Fig. 2:**
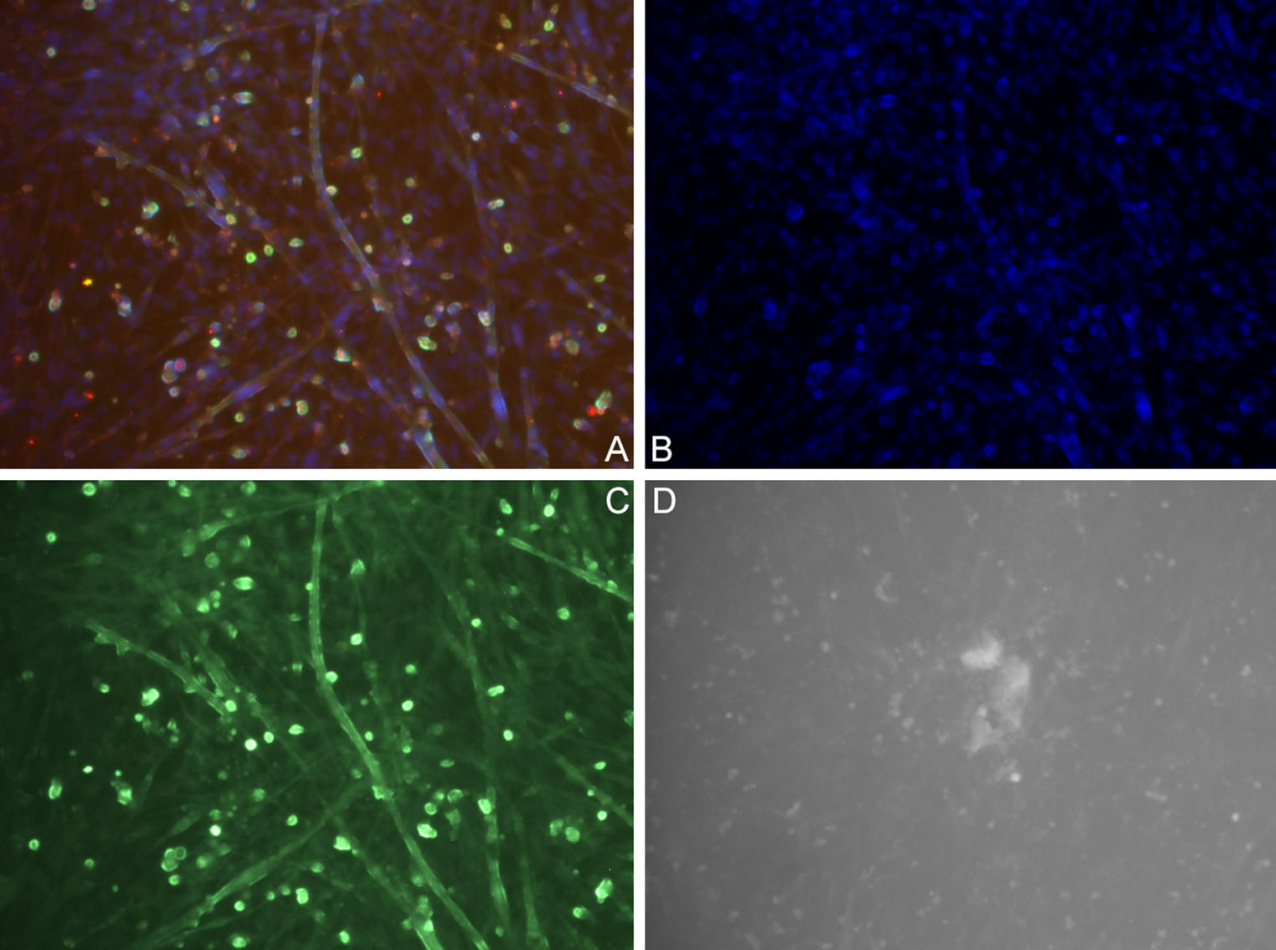
**A**. IF assay showing the cytopathic effect of adenovirus strain JSN-5/10j, IIIp. at 96 h.p.i., with cell nuclei stained blue. **B**. IF of cell nuclei stained blue. **C**. IF of CEF cultures infected with adenovirus strain JSN-5/10j, IIIp. at 96 h.p.i. **D**. IF of CEF SPF, uninfected K- negative control

After CPE was observed, the strains were examined by IFA, and positive results were obtained for both ATCC FAdV-7 and JSN-5/10j. A characteristic effect of fluorescence with different gradations depending on the CPE changes was observed. No fluorescence was observed in uninfected CEFs. The CPE of the JSN-5/10j strain is shown in (Fig. 2A-D). Based on the results of real-time PCR, CPE analysis, and IFA, the examined virus strains were identified as fowl adenoviruses. Molecular analysis was conducted to determine their relationship to other adenoviruses.

### Sequences comparison

The sequences of the standard strains FAdV-7 B-3A, YR36 and eight field strains available in the GenBank database were compared. The results were analyzed for correctness of sequencing and to determine if all nucleotides were identified. The loop L1 regions of the examined strains were compared. This region is positioned between nt 18,649 and nt 19,166 in the FAdV-1 CELO genome (GenBank U46933.1), which corresponds to nt 361-878 of the hexon gene sequence (GenBank AAC54912.1). A 543-bp fragment, comprising the whole nucleotide sequence of loop L1 and flanking fragments of the pedestal (P1) region was used for analysis. Conserved regions located outside loop L1 are the P1 regions, and parts of these were also included in the analysis of loop L1. The terminal regions at nt 10-20 were removed due to sequencing mistakes that could have influenced the results of the analysis. All nucleotide sequences characteristic for loop L1 of aviadenovirus strains were verified.

The nucleotide sequence of strain JSN-5/10j was compared to those of strains B-3A (AF339922), and YR36 (AF508955), and differences between JSN-5/10j and B-3A were found at only four positions. A transversion of thymine to adenine at position 85 (85T>A) resulted in an amino acid change from serine (S) to threonine (T). The second mutation, at nt 102, was a change from T (thymine) to C (cytosine) that did not change the amino acid at that codon. The third mutation at nt 447 was probably caused by a sequencing error that theoretically could change the amino acid sequence. The fourth mutation, at nt 471, was an A>G transition that did not affect the encoded amino acid.

**Fig. 3:**
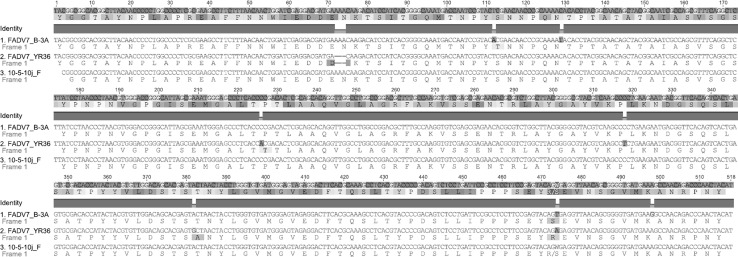
172 aa amino acids from a nucleotide sequence of 518 nt of adenovirus JSN-5/10j strain and reference strains B-3A ATCC VR-832 (AF339922), and YR36 (AF508955)

**Fig. 4:**
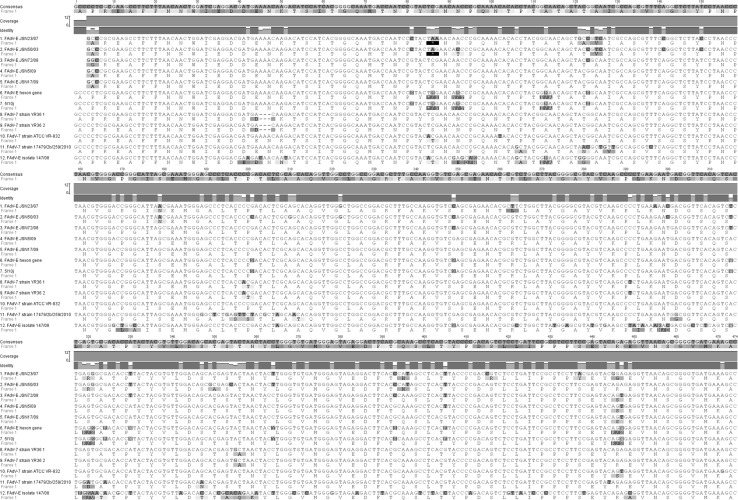
Nucleotide sequence alignment of strain 5/10j and FadV-7 hexon gene fragments. Amino acid sequences of translated sequences are shown under each nucleotide sequence. The consensus sequence is shown at the top of the figure. Disagreements with the consensus sequence are shown in color (color figure online)

Four differences were found between the sequence of JSN-5/10j and that of reference strain YR36. There was a deletion at nt 45-47of YR36 that did not cause a frameshift. A C>A transversion was found at nt 199, resulting in an amino acid change from P (proline) to T (threonine). At nt 201, a transition of C (cytosine) to T (thymine) had no influence on the amino acid sequence. At nt 355, a transition of A (adenine) to G (guanine) resulted in a change from T (threonine) to A (alanine) (T70A). These results are summarized in Figs. 3 and 4.

The pairwise identity of the examined sequences was 94.4%, as expected for strains of the same genotype. The most diverse strain was FAdV-E 147/08, whose identity to other strains was not higher than 86.4%. The JSN-5/10j strain was most similar to FAdV-E (accession number KP879219.1, no serotype information available in GenBank), with 98.4% pairwise identity. Details are presented in Table 1.

**Table 1:** Codon nucleotide composition in the examined region of loop L1

Serotype	T (U) (%)	C (%)	A (%)	G (%)	Examined region (nt)	T-1 (%)	C-1 (%)	A-1 (%)	G-1 (%)	pos #1 (nt)	T-2 (%)	C-2 (%)	A-2 (%)	G-2 (%)	pos #2 (nt)	T-3 (%)	C-3 (%)	A-3 (%)	G-3 (%)	pos #3 (nt)
FAdV-1	18.1	30.1	23.9	27.9	519.0	13	22.3	29.1	36.0	175.0	24	27.3	31.4	16.9	172.0	17	40.7	11.0	30.8	172.0
FAdV-2/11-D	19.7	30.3	26.1	23.9	522.0	16	16.7	31.0	36.8	174.0	23	32.2	31.0	13.8	174.0	21	42.0	16.1	21.3	174.0
FAdV-4	16.8	34.4	23.5	25.3	506.0	18	18.1	31.0	32.7	171.0	19	35.1	31.5	14.3	168.0	13	50.3	7.8	28.7	167.0
FAdV-5	19.8	30.1	26.0	24.1	531.0	15	16.4	37.9	30.5	177.0	21	35.6	28.2	14.7	177.0	23	38.4	11.9	27.1	177.0
**FAdV-7**	**17**.**9**	**30**.**9**	**26**.**5**	**24**.**8**	**525**.**0**	**15**	**20**.**0**	**33**.**7**	**30**.**9**	**175**.**0**	**22**	**32**.**0**	**29**.**7**	**16**.**6**	**175**.**0**	**17**	**40**.**6**	**16**.**0**	**26**.**9**	**175**.**0**
FAdV-8a	17.9	30.9	24.2	27.0	525.0	14	17.7	32.6	35.4	175.0	22	30.9	28.6	18.3	175.0	17	44.0	11.4	27.4	175.0
FAdV-8b	18.1	29.3	27.0	25.5	525.0	15	18.9	30.9	34.9	175.0	21	31.4	31.4	16.0	175.0	18	37.7	18.9	25.7	175.0
Average	18.3	30.8	25,.	25.5	521.9	15	18.6	32.3	33.9	174.6	22	32.1	30.3	15.8	173.7	18	41.9	13.3	26.8	173.6

FAdV-7 - 5/10j strain which was chosen for the study is highlighted in bold

T (U), thymine (uracyl); C, cytosine; A, adenine; G, guanine. Nucleotide occurrence in the examined region of the L1 loop and in the first (T-1, C-1, A-1, G-1), second (T-2, C-2, A-2, G-2), and third (T-3, C-3, A-3, G-3) position of the codon

**Fig. 5:**
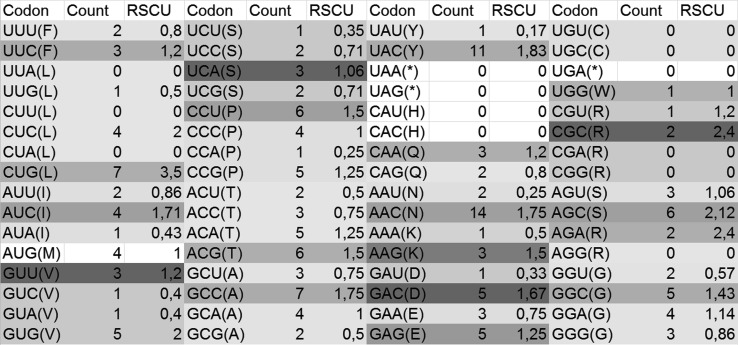
RSCU for adenovirus strain JSN-5/10j. Darker shading indicates a higher RSCU value

**Fig. 6:**
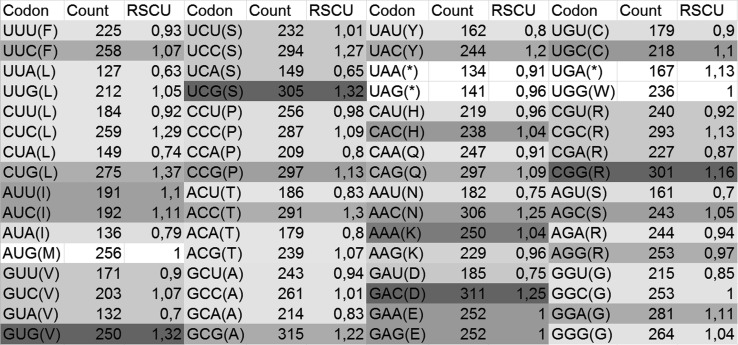
RSCU for adenovirus genome FAdV-1. Darker shading indicates a higher RSCU value

Analysis of the relative synonymous codon usage in the hexon gene region revealed differences in these regions depending on the strain and serotype. The results are presented in Figs. 5 and 6.

### Codon usage

The codon usage in the loop L1 region of the hexon gene was examined, and it was found that C (cytosine) was the most frequent nucleotide for each serotype ranging from 29.3 to 34.4.% G (guanine) appeared most often in the first position of the codon in all examined serotypes, and the percentages were estimated to be between 36.8 and 32.7, except for serotypes FAdV-5 and FAdV-7, where A (adenine) appeared most often in the first codon position (33.7%-37.9%). In the second position of the codon, C (cytosine) appeared most often in serotypes FAdV-5, FAdV-7, FAdV-8a, and FAdV-8b, and the estimated values were between 30.9% and 35.6%. In serotypes FAdV-1, FAdV-4, and FAdV-2/11 (FAdV-D) A (adenine) appeared most often (31.0% - 31.5%) in the second position of the codon. C (cytosine) was most frequent in the third position of the codon in each examined serotype. The values for cytosine were between 38.4% and 50.3%. The results for serotype FAdV-7 and a comparison to other serotypes are presented in Table 2.

**Table 2:**
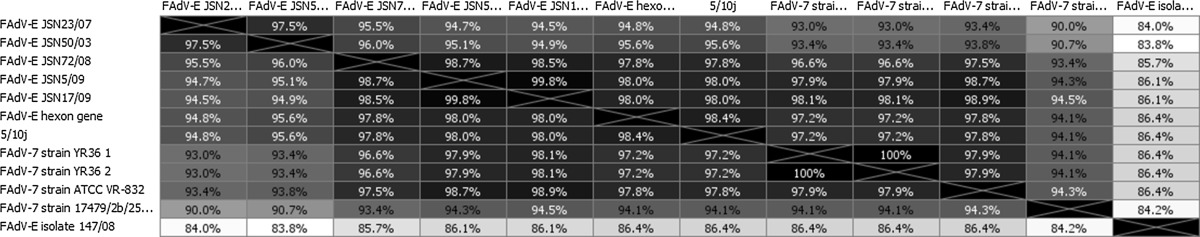
Percentage of identity between strains from one genotype (FAdV-7)

## Discussion

The study was based on analysis of nucleotide and amino acid sequences of the loop L1 region of the hexon gene of an adenovirus field strain isolated from sick chickens. Molecular diagnosis was based on a previously developed and optimized real-time PCR and was focused on FAdV detection [23]. Primers were based on the loop L1 region of the hexon gene. Loop L1 is the region of the adenovirus genome where sequences specific for all fowl adenovirus serotypes are located. After confirming the presence of FAdV-7 DNA, the next step of the study was the observation of a characteristic CPE in CEF cultures and performing IFA with infected cells. Most authors recommend cell culture as the best system for adenovirus cultivation [8]. The degree of CPE and the speed with which it develops are serotype/species dependent and therefore can be useful in strain identification.

In the present study, characteristic CPE was observed upon infection with both the field stain under investigation and reference strains. The CPE was characterized by the occurrence of small, round cells, which were observed starting from 18-24 h postinfection, suggesting that all examined strains belonged to the same serotype.

For the confirmation of the examined adenovirus strain, CPE analysis and IFA were performed. The results of real-time PCR, CPE, and IFA confirmed those of previous examinations. In the next step of the study, serotype/species molecular classification was carried out.

In most cases, researchers target hypervariable regions and HVR1-4 flanking the conserved regions for studies on the taxonomy and antigenic properties of adenoviruses [10, 16, 21, 23–26, 31]. The potential effects of mutations on the hexon protein structure were examined. Nucleotide sequences were translated into amino acid sequences (aa), and a region of 172 aa was analysed. As many authors have suggested [7, 9, 14], the most important mutations are those in the first and second codon positions, because these mutations are more likely to result in an amino acid change affecting the structure and function of the protein. Nucleotide sequence analysis indicated that different codons can code the same amino acid but some of them are preferred. Codon analysis of the loop L1 region of the hexon gene indicated differences in codon preference patterns between adenovirus strains representing diverse serotypes. In most strains the codon CUG is the preferred one for leucine (data not shown); however, in the strains from the FAdV-2/11 and FAdV-4 groups, the preferred codon for leucine is CUC, with Relative Synonymous Codon Usage (RSCU) values of 2,4 and 3,43 respectively. In the case of serotype FAdV-4, CUC and CUG are used at the same frequency. In serotypes FAdV-1, FAdV-4, FAdV-8a, and FAdV-2/11, the preferred proline codon was CCC; however, in serotypes FAdV-5, FAdV-7, and FAdV-8b it was CCU.

The codon CUG codes for lysine. The percentage of GC for different serotypes/species differs, with an average of 56.3, similar to what has been reported by Raue et al. [26]. Genes with stronger transcription often have a higher percentage of GC basepairs [7, 14]. Loop L1 is the most important site of sequence diversity in the hexon protein [5, 25, 26, 30].

Theoretically every codon could appear with equal frequency; however, one codon can be preferred among others coding for the same amino acid [7]. These preferences can appear in genes that are strongly expressed [2, 14]. Optimal codons can lead to more precise and faster translation. This is important for proteins that are synthesised in great quantity [9].

Four amino acid substitutions were found in the L1 region, affecting individual strains only. However, the strain FAdV-JSN-5/10j did not contain any amino acid substitutions compared to the reference strains and the sequence identity between strain JSN-5/10j and reference strains B-3A ATCC VR-832 and YR36 was 97.2%-97.8%.

In conclusion, molecular characterization of certain serotypes, such as serotype FAdV-7, can lead to development of new methods for strain differentiation and help clarify the pathogenic role of FAdV infections in poultry.
